# The Long Fox Memorial Lecture: Ten Years' Progress in Surgical Treatment

**Published:** 1929

**Authors:** A. Rendle Short

**Affiliations:** Surgeon to the Bristol Royal Infirmary


// The Bristol
Medico-Chirurgical Journal

"*Scire est nescire, nisi id me
Scire alius sciret.*"

WINTER, 1929.

### THE LONG FOX MEMORIAL LECTURE:

DELIVERED IN THE PHYSIOLOGICAL LECTURE THEATRE OF THE UNIVERSITY ON OCTOBER 22ND, 1929.

THOMAS LOVEDAY, LL.D., *Vice-Chancellor of the University of Bristol, in the Chair.*

BY

A. Rendle Short, M.D., F.R.C.S.,
*Surgeon to the Bristol Royal Infirmary,*

ON

### TEN YEARS' PROGRESS IN SURGICAL TREATMENT.

---

I desire to express the fullest and sincerest gratitude to those who have done me the honour to invite me to deliver the Long Fox Lecture, and also to those who have been good enough to form an audience. And this for two reasons. First, I heartily agree with a sentence which I heard fall from the lips of Lord Moynihan, that no honour which comes to a man is

U

more acceptable than one that he receives from his own professional colleagues. The other reason is, that I am among the few who have a personal recollection of the great physician whom we gather to honour, and it is a very happy one. Soon after I joined the Bristol Medical School he invited me, with other "freshers," to his house up by Clifton Parish Church, and after coffee and cakes said a few words to express the hope that we would not only be good students, but that we would remember that character matters more than learning. It was the only occasion on which I saw him. He impressed me then, and since, as a splendid example of the best type of the Victorian physician. His writings show keen observation and wide scientific reading. One may know the man from a quotation on the front page of one of his books, in Greek from the Septuagint: "Lo, these are parts of His ways, but how little a portion is heard of Him ?" It may not be known to all present that he and his father and grandfather before him served the public of this city as physicians to the Bristol Royal Infirmary for nearly a century. The first Dr. Edward Long Fox was elected in 1786, and the one in whose memory this lectureship was founded held office from 1857 to 1877.

The remembrance of these doctors of a former generation turns our minds naturally to the differences in practice then and now, so the subject which I have chosen is "Ten Years' Progress in Surgical Treatment." Ten years ago was the commencement of a new epoch. The war was over, the young members of the profession were taking up their permanent work, and fresh minds were to concentrate on the necessarily rather neglected problems of surgery in civil practice. Ever since the world demobilized there has been a voluminous output of surgical literature, good, bad and indifferent. It seems desirable that from time to time some attempt should be made to separate the little wheat from the much chaff. That the ten years have seen real and remarkable advances is beyond all question. It would be tedious, nay, impossible, in the time at my disposal to give a bare catalogue of all that might well be called progress. Being compelled to make wholesale omission of much that others would have put in the forefront, I shall adopt the line of including only that which has proved to be of marked value and interest in my own experience. One or two examples may be quoted of new methods that promised well but do not seem likely to survive. It was advised, on the basis of animal experiments, to drain the thoracic duct in cases of peritonitis. This proved useless in practice. Some surgeons resected parts of the sympathetic chain of ganglia to alleviate hypertonus of the limbs. The benefit is not worth the trouble. Quite recently, however, in the Mayo clinic, resection of sympathetic ganglia has been practised for megacolon (Hirschsprung's disease), with very promising results. Dr. Long Fox would have been interested in this. He wrote a book on the sympathetic nervous system in disease.

I think that the outstanding advances in surgery during this period may roughly be grouped under two heads. Chemistry and physics have not stood still whilst the healing arts are advancing, and they have contributed their quota. Real progress has been made by the use of colloid preparations, diathermy, fulguration, massive dosage with X-rays, improved X-ray diagnosis, especially in abdominal conditions, and, above all, radium therapy for cancer. In the second place, there has been a wider application of the most important principle that we must do the least that will be effectual for a patient who is very ill. A sound man will survive heroic procedures which will be fatal when he is in an acutely toxic condition or pulled down by vomiting and starvation. We shall meet numerous examples of improved results by working on these lines. Better judgment of what to do for very sick people has saved more lives than the invention of new and drastic operations. The anæsthetists must take the credit for some of this improvement. The use of gas and oxygen in abdominal cases, very light ether anæsthesia, and the combination of morphia, novocain and an inhaled anæsthetic have undoubtedly carried some patients through whose condition was so bad that they would scarcely have survived an old-fashioned ether or chloroform narcosis. It may be that the new spinal anæsthetic, spinocain, will also prove to be of life-saving value.

I shall not on this occasion, tempting as it is, comment on the new methods of radium treatment for cancer, partly because it is too large and important a subject to dismiss in a brief space, and partly because our experience of it, though eminently satisfactory over a considerable number of cases, is very recent, all within the present year. Nor shall I include advances in the treatment of fractures, nor in orthopædic surgery.

If I do not make reference to the important post-war contributions of Professor Hey Groves, it is not because I underrate their value, but because they lie in a department of surgery in which I have no special experience.

It is very pleasant to record that some of the progress of surgery during the past ten years has been due to my colleagues at the Bristol Royal Infirmary. Until three years ago actinomycosis was a very grave disease. In 1907 I published a study of cases of actinomycosis of the appendix, all of which died after a long, miserable ilness, and that has been the fate of some seen since. In 1926 Mr. Chitty, acting on a suggestion by Mr. A. L. Taylor, advocated the administration of iodine in milk, five or ten drops three times a day, and I can fully confirm its value. A patient of mine with actinomycosis of the jaw responded excellently to this treatment. We, in this district, should also be grateful to Mr. C. F. Walters for his introduction of mercurochrome for a variety of ailments. Its first and best value is for B. coli and other forms of pyelocystitis. It seems to have some power of ascending the ureter and acting even in the pelvis of the kidney, and we have seen numerous cases in which, after the introduction of an ounce of mercurochrome solution into the bladder, the temperature has come down and the frequency of micturition abated. A word of caution is necessary; some patients are sensitive to it, and the first dose ought not to exceed $\frac{1}{4}$ per cent., though 1 per cent. is the optimum therapeutic strength. Also, it is better not to repeat it more than once or twice a week. But it has other virtues besides its antiseptic effect in bladder cases. It can be given intravenously, one ounce of 1 per cent. solution, not boiled, for septicæmia, its high bactericidal power and low toxicity making it peculiarly suitable for this purpose. Last year it seemed to be life-saving in a public school-boy with a fulminating osteomyelitis of the iliac bone and pleuro-pericardial friction. I have seen other cases. It is also very useful for injured or infected joints, and for big lacerated wounds associated with compound fractures.

Still dealing with general surgery, I believe the intravenous or intramuscular injection of 15 c.c. of 30 per cent. sodium citrate solution, introduced in

1922 by Neuhof and Hirschfeld, is of great value in checking bleeding, such as hæmatemesis, or oozing after operations. It acts, as they showed by animal experiment and observations in man, by shortening the coagulation time of the blood, the maximum effect being reached in three hours and waning after twenty-four hours. It is effectual in jaundice, but not in hæmophilia or purpura. It is well also to give hæmoplastin, which has a delayed action and is more effectual after twenty-four hours.

The researches of Professor Leriche on the nerves running in the walls of arteries are of great interest, and enable us to help some patients who were previously beyond our skill. I have used decortication of an artery in a small series of cases. It has been recommended for painful extremities associated with coldness and vascular paralysis ; one case did well, but another was not benefited and came to amputation. He had neuritis leading to a patchy gangrene of the foot. I have also tried alcohol injection of the arterial wall for incipient gangrene in senile cases ; it has relieved the pain, but has not in my hands averted the necessity for amputation later. The theory, of course, is that the deep pain nerves run with the arteries rather than with the main nerve trunks. As soon as the artery is decorticated the vessel is seen to contract so tightly that almost no blood gets through ; after twenty-four hours it opens up widely. The method is advised for the relief of trophic ulcers, but I have not had occasion to use it.

We have become awake to the fact, previously not recognized, that septic infections of the face and lips carry with them a serious danger to life. Two years ago I was asked to see a powerfully-built, athletic man of 21, who had had a pimple behind the ear for a couple of days. It was quite small, rather sloughy, and there was a slightly enlarged lymphatic gland, but he looked very toxic. Next day he died. I have never seen a more tragic case, nor one in which there seemed so little one could do. Two other of my patients within the last ten years have died from a similar cause. It is very bad practice to incise these infected swellings until there is a definite abscess. It is better to trust to fomentations, and when there is a raw surface Morison's paste (desiccated magnesia 30 oz., glycerine 11 oz.) is a valuable application. Some German authorities inject the patient's own blood around the swollen area. Rœder, and also Hamilton Bailey, advise, when the lesion is on the lip, that the angular vein should be tied at the side of the nose, to prevent infection of the orbit and intracranial cavity, but I have not had an opportunity of trying these measures in a really suitable case.

One of the foremost advances of the last ten years, without doubt, relates to the work of Kanavel and others on infections of the hand. One shudders to think how ill these cases used to be treated. Some of the points, of course, are old ; some are new. We find here another application of the law to do the least that will be effective for the very acute case ; when there is, as yet, no evidence of pus-formation in the finger but an angry red streak of lymphangitis runs up the arm, it is dangerous to incise. When pus is thought to be present, whether on the finger or in the hand, the method of opening must be deliberate, using a tourniquet so that we may see what we are doing, and an anæsthetic so that there need be no hurry. If there is pus in a tendon sheath it must be followed up, and it is very necessary to know the exact line of incision which experience shows is most suitable in each case. In particular, when the front of the wrist is involved, it is useless to make cuts over the flexor tendons; they miss the pus and probably infect the sheaths. The incisions must be at the sides, just in front of and along the borders of the radius and ulna, so that the tendons may be approached from behind. The newer work shows that there are two fascial spaces deep in the palm, the mid-palmar space and the thenar space, behind the flexor tendons but in front of the metacarpals. When these contain pus it must be evacuated without damaging the tendon sheaths; the thenar space from the outer side by an incision just in front of the second metacarpal, and the mid-palmar space through the webs of the inner fingers. In these cases the back of the hand is œdematous, and this has led to much fruitless incising, to the great detriment of the extensor tendons.

*Surgery of Thyroid Intoxication.*—Not so many years ago the surgical treatment of exophthalmic goitre was looked upon as hazardous in the extreme. The earlier cases at the Infirmary nearly all died, sometimes with the most distressing symptoms of acute hyperthyroidism. Several perished on the operating table. Nowadays our experience is that these cases can be operated on quite as safely as, shall we say, a gastric or duodenal ulcer, and more safely than an enlarged prostate, and also that they can with fair certainty be almost entirely, if not entirely, freed from their symptoms. In the years 1925 to 1928 I had an unbroken series of thirty-three operations for thyroid intoxication (twenty-three of exophthalmic goitre, ten of toxic adenoma) without a death. This year has been less fortunate.

What are the improvements by which this great change for the better has been effected? First, by a better classification of the cases. There are two types, not sharply demarcated, but with all gradations between them : classical exophthalmic goitre or primary thyroid intoxication, and toxic adenoma or secondary thyroid intoxication. In the former the enlarged thyroid, nervousness, tremor, loss of weight, and tachycardia all come on together, the goitre is uniform, very soft and vascular at first and firm later. Exophthalmos is the rule. Operation is not indicated till the physician has had plenty of time to bring about a cure by rest, remedies and X-rays, but if there are still marked symptoms present after a year operation is generally indicated. We are tending to shorten this period as the safety and benefit of partial thyroidectomy become more manifest. In toxic adenoma or secondary thyroid intoxication there have been rounded adenomatous nodules in the gland for years causing no symptoms, then the above-mentioned signs of thyroid intoxication come on, but usually there is no exophthalmos. If neglected, hopeless damage to the heart muscle ensues. These patients should be operated on without delay, as natural recovery is improbable and a downward course is the rule. A good many patients whose main symptom is persistent tachycardia are found on careful search to have a small nodular thyroid adenoma, easily overlooked, removal of which will cure them. With better classification has come more precision in pre-operative treatment. A short course of Lugol's iodine, aided by rest in bed for a week or two, will, as a rule, greatly improve the heart and general symptoms in a case of exophthalmic goitre, and make them much safer subjects for operation, so that the acute intoxication during the first twenty-four hours with high temperature, delirium and violent tremor is seldom if ever seen now, and thus the greatest terror of thyroid surgery has disappeared. In secondary cases iodine seldom does any good and may be harmful. Another forward step is our modern reliance on the graded operation and on better technique. It is necessary to take away seven-eighths of the total gland substance to abolish symptoms with practical certainty, but that is often too much to do at one sitting. The first intervention is therefore usually a hemi-thyroidectomy. If the patient is very ill it may be wise to commence by tying the superior thyroid vessels and no more, but of late surgeons who specialize in these cases have become more venturesome, and, I think, rightly. A few months after the hemi-thyroidectomy the greater part of the second lobe is removed. I have often found, however, that there has been such a degree of improvement, that although it falls short of a complete cure, it is not worth the small risk and inconvenience of a further operation to rid the patient of the remaining symptoms. The principal risk nowadays is hemorrhage about five hours after the operation has been completed, and to avert this far greater precautions ought to be taken than are usual in the surgery of less vascular regions. Big vessels should be doubly ligated, three-cast knots used, all but the simplest ties should be secured by stitching, the anæsthetist should let the patient come round and strain before the wound is closed, and a most careful post-operative watch should be kept for the first twelve hours. I have only once had any anxiety about any patient who has come safely through that period, and she proved to be suffering from a latent pyelitis. Other surgical risks, such as tetany and division of the recurrent laryngeal nerve, may be avoided by leaving a portion of the thyroid close against the trachea on each side.

*Cervical Rib.*—The condition usually called cervical rib requires some elucidation. It is quite true that a patient presenting the well-known symptoms of pressure on the cords of the brachial plexus, especially the inner cord, may have a genuine rib articulated to the seventh cervical vertebra. More often, what is called a cervical rib is nothing but an enlarged transverse process, and it is very doubtful whether it could exert any mechanical pressure on the nerves. Not infrequently one sees a case where the nerve symptoms are just such as are associated with cervical rib, but the X-ray of the vertebral column shows no abnormality at all. Stopford described a series of examples of this, and attributed the pressure to the first thoracic rib. His patients were cured by detaching the scalene muscles from the rib and resecting a portion of the rib itself. Law maintains that the trouble is due to tense fibrous bands. More recently Adson and Coffey blame the scalenus anticus, and say that all that is necessary, even if a genuine cervical rib is present, is to divide this muscle. I have had a number of cases entirely confirming these views, in which the symptoms were at once relieved, either by resecting a portion of the first rib or by cutting through the scalenus anticus. In one case the patient was able within a day or two to do needlework, which had previously been impossible. The skiagram of her neck, as in other cases under my care, showed no abnormality of the bones.

The practical deduction is that we are now in a position to afford relief to cases of pain down the arm, or wasting of the hand muscles, even if there are no bony abnormalities.

*Empyema.*—Speaking in general terms, the lessons of war surgery have not been of as much assistance to civil practice as was anticipated, and as the public imagine. Progress in the treatment of empyema may, however, be directly attributed to experience gained in the handling of wounds of the chest. Up till 1914 the majority of surgeons intervened at once as soon as the needle showed pus, or even a turbid fluid, in the pleural cavity and put in a drain, which, of course, admitted air and germs. We learned in the war that wounds opening the pleural cavity give rise to great shock and distress, owing to flapping of the mediastinum, which is dragged across to the sound side ; this distress is promptly relieved by covering the hole in the chest wall. Experience also showed, especially amongst troops in America during the influenza pandemic of 1918, that early operating for empyema, before the mediastinum is fixed by adhesions and especially in cases in which the whole pleura is full of fluid and the lung is collapsed entirely, was associated with a terrible mortality, about 60 per cent.

Thus, there are two lessons to be learned. First, to postpone opening until the mediastinum has become fixed by adhesions and thickening of the pleura. Secondly, as far as is compatible with the surgical principle that to cure an abscess we must relieve the tension, to use a closed method after the operation. The practice which I follow, and which has in every case given a good result and healing within a few weeks (one case treated by the old method, by way of contrast, took a very long time to close), is this. When the effusion is due to some other cause than the pneumococcus, or when it fills the whole or nearly the whole of one side of the chest, it is treated by aspiration, repeated once or twice if necessary, until it becomes localized ; so time is given for adhesions to develop. Then, except in the improbable event of the fluid clearing up entirely, the chest is opened, perhaps after a week of this preliminary treatment, a piece of rib is resected, the pus and curdy clots evacuated, and a rubber tube at least a foot long is inserted, only an inch or less being inside the pleura. The pleura, the muscles and the skin are sewn tightly around the tube so that it is air-tight. The tube is clamped near its farther end, say ten inches from the chest. Several times a day the end of the tube is put under lotion, the clamp taken off and the patient told to cough and breathe deeply. The clamp is put on again at the end of a cough. By this means the lung is brought out to meet the chest wall and kept there. It is true that after four or five days—most valuable days—pus will usually leak around the tube, and it can be cut off close to the chest in the old-fashioned way, but by that time the lung will have expanded unless the empyema is of long standing. If the cavity is very septic washing out with eusol is effectual.

When empyema is localized from the first, and follows pneumonia, there is no need for preliminary aspirations ; the tube may be inserted as soon as the diagnosis is made.

*Cancer of the Œsophagus.*—This has always been, and to a reduced extent still is, one of the most hopeless propositions the surgeon has to deal with, but it has been the subject of many therapeutic experiments of late years, and sometimes something can be done. Of radical removal I have no experience, though I have seen successful cases in cancer not far below the cricoid. Deep X-rays, in a minority of cases, may prolong life and improve the power of swallowing, but I have not seen it. Very likely radium will be the method of the future, but at present inserting it into the lumen is useless, and stabbing it into the wall of the œsophagus around the stricture carries a prohibitive mortality. Gastrostomy, as it used to be performed, was a very dismal method of attempting to give relief, and if the tube was left out because the patient had some temporary power of swallowing still, the orifice quickly closed and rendered it ineffective. There is a good technique now available, which I use and thoroughly approve of, introduced by Quick and Martin, which brings a tube of gastric mucosa through the rectus abdominis and provides a channel which neither leaks nor closes, although it may not be used even for months.

The flexible wire tubes introduced by Souttar are excellent when they can be placed just in the right position and when a fairly large tube fits firmly, but it is not always easy to secure this, and sometimes they lie entirely above the growth, or slip through into the stomach. I think they are at their best when the cancer lies close to the cardiac orifice, as shown by the X-ray. I have treated several cases of this type by an exploratory laparotomy, opening the stomach, dilating the stricture by retrograde catheterism, starting with Harrison's whip and going on to œsophageal bougies, drawing down a full-size Souttar tube and seeing that it fits correctly, just projecting into the stomach lumen. The stomach is then closed. The patient will be able to swallow soft food through the tube. If this procedure is not possible, one can at least do a Quick and Martin gastrostomy, so that the intervention is not wholly in vain.

When the obstruction is farther up, I find most relief from our colleague Mr. John Wright's diathermy knife, provided that one can pass a fine bougie through the stricture. One is painfully aware, of course, that none of these methods of treatment offers any hope of a real cure ; all are palliatives.

*Abdominal Surgery.*—Some general principles have come to the fore which have considerably reduced the risks of abdominal surgery, and increased the patient's comfort in the post-operative period. One such principle, that of doing the least that will relieve the immediate situation in very sick patients, has already been mentioned.

The old bugbears of abdominal surgery have been what the Americans expressively call gas-pains, ileus and chest complications. There is no doubt that some of these were due to over-preparation. Starvation and purgation before operation and a prolonged course of slops with more purgation after, were enough to upset the healthy, and how much more when a laparotomy was added ? Now, all these measures are reduced to a minimum. We have learned that if the intestines are pulled or pressed or their surfaces rubbed with gauze they will revenge themselves by semi-paralysis afterwards. The greatest gentleness must therefore be observed, and rubber-faced sheeting is better than taped swabs made of gauze. If intestinal paralysis does occur, and is due neither to mechanical kinks nor local peritonitis, we have learned that the combination of physostigmine and pituitary extract is more effective than either by itself in securing an action of the bowels.

Chest complications after abdominal surgery have been particularly frequent in stomach and gall-bladder cases, and are due to several factors. Ether anæsthesia is one, and the present tendency to use gas-oxygen has, in my experience, helped a good deal. Another factor, probably more important, is reflex palsy of the diaphragm owing to painful sensations derived from the cut abdominal wall, leading to collapse of the base of the lung. Now, when we reflect on this, it is evident that, seeing we cannot do without an incision in the abdominal wall, we should at least take steps to cut as few nerves as possible, and sew up with as little tension as we can. All longitudinal incisions are bound to cut many nerves, and there is often great tension in suturing, especially with the paramedian. Of late years I have used the Sloan incision for upper abdomen work, with the greatest possible satisfaction. It cuts no muscles and the fewest possible number of nerves, and always comes together without tension. It is difficult to see how incisional hernia can follow. The post-operative comfort of the patient, and the facility with which he moves about in bed the day after the laparotomy, and the improbability of adhesions developing afterwards are all strong points in its favour. Briefly, the method is to make a median longitudinal skin incision down the linea alba; then a double paramedian through the anterior sheath of the rectus on each side; then a transverse cut, behind the recti, through the posterior rectus sheath and peritoneum. This is the level at which the important nerves run, but a transverse cut obviously saves many which a vertical incision would divide. Next best to the Sloan is a simple transverse incision. For operations on the ascending colon, an oblique incision in the line of the nerves, but dividing the muscles—the external oblique in the line of its fibres, the internal oblique across them—has the same virtue of sparing many nerves from section. By the use of these devices post-operative bronchitis and pneumonia have been far less common in my practice than they were a few years ago. One may still have to use vertical incisions, of course, when a complete exploration is necessary.

*Gastric Surgery.*—The main progress here, in my opinion, is in diagnosis rather than in treatment. The perfecting of barium skiagraphy in stomach cases has been of the greatest possible benefit and comfort to us. Stomach cases used to fill me with despair on account of the uncertainty of diagnosis and the frequency of vain operations; to-day we can nearly always pick our cases suitable for surgery with reasonable confidence.

Of course, surgical treatment has not stood still when dealing with the stomach. The tendency has been to do a bigger operation for gastric and for duodenal ulcer, either by combining excision of the ulcer with a gastro-enterostomy, or by resecting the pyloric half, or rather more, of the stomach. At first gastric ulcers only were excised, now some would remove duodenal ulcers also. The original argument for the larger operation was that gastric ulcers very commonly become malignant. The general opinion, which my experience heartily supports, is that this takes place in only a minority of cases. It may, however, be very difficult to tell, on the table, whether an ulcer is innocent or not, and on two occasions the pathologist has returned an ulcer as cancerous which I was quite sure was innocent. A further argument in favour of the removal of a large part of the stomach is that the clinical results are better, in that there is less danger of recurrent ulcer, and especially of that dreaded complication, gastro-jejunal ulcer. This is stated to occur after about 2 per cent. of gastro-enterostomies. On the other hand, there are a few cases on record of intense anæmia following the big partial gastrectomy; I have had one in my own practice, but have not yet seen gastro-jejunal ulcer in any patient operated on by myself, in spite of careful following up. My own feeling, therefore, is in favour of local excision with gastro-jejunostomy for small and medium-sized gastric ulcers; simple gastro-jejunostomy for irremovable pyloric

V

and for duodenal ulcers; and partial gastrectomy for large, deep, adherent gastric ulcers. If they cannot be removed, cholecyst-gastrostomy may be done to neutralize the acid juice, but whether this gives more relief than a simple gastro-jejunostomy proximal to the ulcer is a moot point.

Various small improvements all taken together have reduced the risks of a straightforward gastro-enterostomy considerably in my practice. These include gas-oxygen anæsthesia, the Sloan incision, placing the anastomosis at the greater curvature of the stomach, taking off the clamps just before finishing the inner stitch to ensure that there is no bleeding, using an interlocking suture to leave no vessels uncontrolled, tying off any large feeding arteries on the stomach wall after finishing the anastomosis, and leaving a piece of rubber dam inside the lumen of the anastomosis to avoid adhesion of the anterior and posterior lips thereof and to straighten out the channel. Something has thus been done to combat the three main risks, pneumonia, bleeding and post-operative vomiting.

*The Surgery of Intestinal Obstruction.*—We are not often in a position to mark statistically the advance of surgery, but we can do so here. Several large bodies of figures, both British and American, show that during the ten or twenty years preceding 1907 the mortality for all forms of acute obstruction (not hernia) was about 60 per cent. The large composite tables of figures for seven hospitals, including the Bristol Royal Infirmary, presented at the 1925 meeting of the British Medical Association, show 1,655 cases with a death-rate of 32 per cent. No doubt the reduction can and will be carried farther still, the general practitioner co-operating by speeding up his diagnosis, and the surgeon by remembering the maxim to do the least that will be effectual for a very sick patient. It is to be hoped that no one now waits till the patient has fæcal vomiting before thinking of this grave condition.

Progress has been made by the introduction of the operations of " blind cæcostomy," or of jejunostomy under a local anæsthetic if the block is in or above the cæcum, leaving the removal of the cause till a more favourable occasion. Of course, these are only emergency procedures, and if the patient is not toxic it is better to explore the abdomen properly under ether or gas-oxygen, with some novocain into the muscles. In either case we now have certain adjuvants. It appears probable that the fatal toxin in these cases is allied to that of gas gangrene, and derived from *B. perfringens* in the bowel. There is an antitoxin on the market against this, and experience at St. Thomas's Hospital goes to show that the injection of 80 c.c. into the buttock in cases of intestinal obstruction with toxæmia, or of peritonitis, may be life-saving. I have used it on a good many occasions, and am impressed with its value. Another valuable aid in such cases is an intravenous injection of hypertonic saline, *e.g.* 100 c.c. of a 5 per cent. solution, to be repeated if necessary. Patients with toxæmia from intestinal obstruction show a deficiency of chlorides in the blood. Of bile and choline, which are also recommended, I have no experience. We now recognize that in cases of pelvic peritonitis, as, for instance, after appendicitis, the condition often passes after a few days into a frank intestinal obstruction due to kinking and paralysis of the coils of ileum, and the treatment should be on the lines just described. I have saved several patients thereby whom I

should have lost, nominally of peritonitis, really of obstruction, in former years.

The exact nature of the poisoning in intestinal obstruction is in doubt. It is said to be a proteose by some investigators, and is identified with histamine by others. It seems clear that the fatal absorption takes place in the duodenum and upper jejunum, and that such absorption is enhanced after ether anæsthesia because of the quiescence of the bowel afterwards, hence the value of jejunostomy under novocain.

I believe the procedure introduced by Stiles, of making a temporary cæcostomy as a routine when resecting a cancer of the left colon or the sigmoid loop, is of great value. Better still is a preliminary cæcostomy a week or two before the main operation. I am also very well pleased with the device advocated at the Mayo clinic, of keeping the patients under morphia and giving fluids subcutaneously, but not by mouth, after a hemicolectomy for cancer of the cæcum or right colon.

*Appendicitis.*—In two particulars there is a change in our treatment of appendicitis. As to the wisdom of immediate removal of the appendix, if possible, before perforation, or as soon after as may be, in acute febrile cases, there can be no question. But a good many surgeons in this country, and more in America, have come to believe that there are occasions when it is wiser to hold one's hand for a time. No universally applicable rules can be laid, each case must be judged on its merits, and there is room for difference of opinion ; but I would suggest that when the patient is first seen on the third, fourth or fifth day of a severe attack and the appendix has already perforated and there is extensive peritonitis with marked toxæmia, it is better to content oneself with a suprapubic drain into the pouch of Douglas inserted under local anæsthesia, and then to follow the Ochsner lines of medical treatment, that is to say, the Fowler position, Murphy proctoclysis, starvation (giving not even water by mouth) and small doses of morphia hypodermically. The appendix may be removed later. Again, when there is a large swelling in the right loin or iliac fossa, usually after the fifth day, one may wisely wait a few days if it is ill-defined, not very tender and the patient not toxæmic, because such swellings are likely to clear up. If the swelling becomes well-defined, tender or pointing, usually after a week or more from the onset, it needs opening, but that is not the time to embark on a surgical struggle to find and remove the appendix. Quite likely it has been entirely destroyed; if it gives further trouble it can be taken out subsequently. These measures are not heroic; we may feel proud of our skill in getting out the appendix that everybody else would have left, but it does not follow that because we can do a thing therefore we ought to do it. For the very sick man, do as little as will tide over the situation.

The other change is in the frequency with which we diagnose and operate on chronic appendicitis. Far too many appendices used to be taken out without relieving the patient's pains. We now recognize that, in addition to genito-urinary sources of trouble, pain in the right iliac fossa may be due to a dropped distended cæcum or to mesenteric lymphadenitis. A young woman who has a dragging pain at McBurney's point all day and every day, but has never had an acute or febrile attack and is relieved by recumbency, is not likely to be cured by losing her appendix. Barium skiagraphy will probably show a large, low, slowly-emptying cæcum with general visceroptosis. In true chronic or relapsing appendicitis which is likely to benefit by surgery there are well-spaced-out brief periods of pain and longer periods of freedom.

*Mesenteric Lymphadenitis.*—It has been recognized for many years that enlargement of the mesenteric glands, usually but not always tuberculous, is exceedingly common, especially in children, but it is only of late years that much attention has been paid to the symptomatology. As a matter of fact, the clinical picture may conform to quite a number of different types: there may be persistent fever, or a palpable lump, or even perforation. A frequent and very suggestive history is that a child gets attacks of sharp abdominal pain which come on without cause, and may be only for a minute or two or may last several days; there are no other symptoms, and physical examination reveals nothing. In adults the attacks may be very violent and simulate renal colic. If the pain is in the right iliac fossa a strangely-close mimicry of appendicitis may be seen, the temperature and pulse may be raised, and there is vague tenderness at or near McBurney's point. It is almost certain, even if the true nature of the case is suspected, that the surgeon will not be sufficiently confident that appendicitis can be left out of the reckoning to refrain from operating. The error is not a serious one, as it will very probably be found that the inflamed lymph nodes are few in number and capable of removal, so trouble in the future is avoided.

*Gall-bladder Surgery.*—We are in the happy position of being able to report great progress in this field. The physiology of the gall-bladder is better understood :

we know that its function is largely absorptive, and that the bile standing in it becomes concentrated. Pathology has advanced. There were two schools, one of which taught that gall-stones were the result of chronic inflammation ascending the bile-ducts into the gall-bladder, causing its mucosa to shed out cholesterin ; the other maintained that the bile is often sterile and that stones are associated with and due to too much cholesterin in the blood. The situation seems to be clarifying as follows : the primary trouble is, often if not always, an infection of the liver. This soaks through to the wall of the gall-bladder (which is more frequently found to contain germs than the bile). Like most mucous membranes, the inflamed mucosa of the gall-bladder excretes cholesterin. Some is absorbed, especially during attacks called "cholesterin showers," and at these times, but not between, the blood shows hyper-cholesterinæmia. The inflamed mucosa may be choked with cholesterin, giving the so-called "strawberry gall-bladder." Later, the germ infection may die out, but the masses of cholesterin, now welded into stones, remain. Re-infections may occur, and give rise to suppurative cholecystitis. There is a not uncommon form, in which a single white stone, usually in a germ-free bile, blocks the neck of the cystic duct, and causes "hydrops," the gall-bladder being tensely filled with white bile.

Methods of diagnosis have advanced. We can now visualize the gall-bladder by means of cholecystography. The functional capacity of the liver, on which the safety of surgery depends, may be estimated by the lævulose and other tests. The Rehfuss tube enables us to obtain duodenal contents and to bring down an efflux of bile by means of magnesium sulphate, which may be examined for pus and germs.

An important addition has been made to medical therapeutics by Hurst's discovery of the possibility of giving hexamine in 100-grain doses, guarded by alkalies, to disinfect the liver, bile and bile-passages. The liability to bleed in jaundiced cases can be controlled to some extent by intravenous injections of calcium chloride.

Surgery has profited by all these extraneous aids. It is evident that removing the stones, by itself, will not relieve the symptoms associated with cholelithiasis, even if no more stones form, because the mucosa of the gall-bladder is probably infected primarily, and will continue to poison the patient and give rise to reflex dyspeptic trouble. Whenever possible, therefore, we now perform cholecystectomy. Done at the proper time, it is little if any more dangerous, and the end-results are unquestionably better. On the other hand, we now realize what a serious factor the accompanying liver infection may be. Most of the deaths, often rather mysterious, that used to follow operations on the biliary system, were probably due to liver failure. Here is a pre-eminent indication for dealing gently with a very sick man. It is my practice, of late years, to wait at least a week after an attack of gall-stone colic, especially if there was fever, before intervening, using liver function tests if in doubt. If one is compelled to operate during an attack, which may happen if there is pus in the gall-bladder, it is wise to use a morphia-gas-oxygen-novocain anæsthesia, to open the abdomen by a transverse incision just over the gall-bladder, and to content oneself with evacuating the stones and pus, and draining. The value of the modern change of practice may be judged by the fact that at the Bristol Royal Infirmary from 1900 to 1912 the death-rate in 84 cases was 13 per cent., and of 59 followed up only 52·5 were classed as cured, and 15 per cent. were unrelieved. Between 1920 and 1929 I have operated on 140 cases for gall-stones or for infective cholecystitis. Many were septic ; a few were over 80 years of age, some had stones in the common duct. Six died ; that is a mortality of 4·3 per cent. One death was from apoplexy. In two other patients a cholecystectomy was done under circumstances where one would now think it better to drain. Of the cases in which the later history is known about two-thirds are symptom-free ; the others are improved ; none are unrelieved.

*Hernia.*—It might be supposed that on so humble a subject nothing more remained to be said, and that surgical technique had permanently standardized itself, but fortunately that is less than the truth. The old operation for femoral hernia, tying off the sac just below Poupart's ligament and sewing the ligament down to the pectineus fascia, had a recurrence rate of 30 per cent. at the Bristol Royal Infirmary. The modern procedure is the Lotheisen operation, in which the sac is approached at the neck, in the inguinal canal, and tied off flush with the peritoneum ; then the internal oblique and conjoint tendon are sewn down to the periosteum at the back of the pubes. Of a personal series of 28 cases treated in this way and followed up there was only one in which there was any recurrence, and that one doubtful.

In ordinary cases of inguinal hernia in adults I still use the Bassini method. There is only one common complication, and that is hæmatoma of the scrotum afterwards. To avoid this very annoying, time-wasting episode I drain the wound for 24 hours, and stitch the bottom of the scrotum up on to the skin of the abdominal wall for a day or two. When the gap is large or the muscles feeble Gallie's suggestion, of darning the internal oblique to Poupart's ligament with a strand of fascia lata, is well worth following. Often the coverings of the hernia may be strong enough to furnish us with our suture, and save cutting the leg.

Obturator hernia is rare, and when it occurs we usually find ourselves operating on it from within the peritoneum. It cannot be satisfactorily closed by stitching, and recurrence is the rule. After one such experience, I blocked the canal by inserting a piece of costal cartilage, and covered it in with a purse-string peritoneal suture ; this was quite a success.

When operating for strangulated hernia, it is running an unnecessary risk of cutting the bowel to divide the tight neck from within the peritoneal sac by means of our old friends the hernia knife and the hernia director. They should be superannuated, and the ring divided from without. The old practice of nicking Gimbernat's very important ligament in cases of strangulated femoral hernia should be abandoned, and Poupart's ligament, which can so easily be sewn up again, sacrificed instead. Gimbernat's ligament is not easily repaired, and if incised there is a permanent weakness and enlargement of the femoral ring.

*Enlarged Prostate.*—One of the chief lessons we have learned concerning enlargement of the prostate is the paramount importance of the function of the kidneys, and the serious effects upon them of the back-pressure resulting from straining in micturition, especially if infection is superadded. It did not occur to us until the post-war period that a man might be free from cystitis, getting about his business in apparent good health, alert mentally and physically, and yet his renal functions be on the verge of bankruptcy, so that removal of the prostate would probably lead on to fatal uræmia. Fortunately we are now in a position to detect these cases with a fair degree of accuracy. The polyuria, the uniformity of the specific gravity of the urine which does not show the normal daily variations, the high blood urea, and most valuable of all, the urea concentration test, demonstrating that even after giving 15 grammes of urea the kidneys cannot concentrate it in the urine, are of the greatest assistance to us. When the renal function is but little impaired and there is no cystitis, we may remove the prostate with a risk well below 5 per cent. When these tests show considerable depression of the powers of the kidneys to excrete, the patient may still be brought safely through, in all but a few extreme cases, by a two-stage operation, the first being either cystotomy, or the insertion of a de Pezzer tube. As a rule the kidneys will show some degree of recovery after the back-pressure has been relieved in this way, and if necessary we may wait three months or more in a bad case. Usually a wait of a fortnight is sufficient. With the de Pezzer tube the man can get about his work without leakage, and by following this practice I have been able to remove the prostate safely in patients whose urea concentration test was as low as 0·8 per cent.

When we are dealing with an aged or feeble patient, or a bad surgical risk, there are other means available besides removal of the whole prostate. K. M. Walker's diathermy punch has a distinct field of usefulness when there is a middle lobe projecting into the bladder, and the risks are very small. X-rays, and diathermy applied with a rectal electrode, may give considerable relief, and are to be borne in mind in cases not severe enough as yet for operation, as well as in bad risks.

Cancer of the prostate is rather a melancholy subject, but even here some progress has been made and some hope can be afforded. The insertion of a de Pezzer tube, if it is tolerated well, gives the patient far more relief than we used to be able to afford him. Sometimes we can do more. The prostate may be capable of enucleation, and radium can be left in the cavity. I have had one or two cases which have gone on comfortably for years, and are still free from symptoms. When this is not feasible a combination of methods may give great relief. Early in the year I was asked to see a gentleman who was in great discomfort with a badly-working de Pezzer tube inserted for cancer of the prostate. Radium needles were introduced into it through a perineal incision. Later, part of the growth, in the floor of the prostate urethra, was burned away with the diathermy punch, and the de Pezzer tube removed. The supra-pubic wound healed, and he has been passing water without frequency or difficulty for six months.

*Male Genital Organs.*—I have not sufficient experience to pass an opinion whether the new, spectacular methods for rejuvenating old men by tying the vas or by ingrafting of monkey-gland can be looked upon as substantial improvements and whether they have come to stay. But I am very favourably impressed by the usefulness of the operation for undescended testis in which after freeing the organ, with as little interference with its blood-supply as possible, a hole is made in the septum scroti and the testis put through to the opposite side. It can be relied on to stay there. True, it may not grow to its full size, but no doubt some internal if not external function is preserved, and at any rate the patient is much better pleased than he would have been with unilateral castration, or restoration of the testis within the abdomen.

Operations on the urethra, both for impassable stricture and for traumatic rupture, are in my judgment greatly assisted, both at the time and afterwards, by a suprapubic cystotomy. At the time, this allows of retrograde catheterism, and makes the otherwise difficult search for the proximal end simplicity itself. In rupture cases, an accurate end-to-end suture can now be carried out. The supra-pubic drainage helps to keep the urine from the site of healing, and both saves time during convalescence and limits the danger of stricturing afterwards. It will usually be necessary in impassable stricture cases to tie in a catheter for a few days. In traumatic cases this is better avoided except, as Hamilton Bailey has pointed out, when the prostatic urethra is torn ; if the catheter is omitted here the bladder is apt to fall back and render the urethra discontinuous ; also, the risk of stricturing is slight in this situation.

*Rectal Surgery.*—Of late years the operation of ligature for piles has largely been replaced, when the piles are within the sphincter, by injecting with carbolic, to the great saving of the patient's time. This is a real advance, and in my considerable experience very satisfactory.

The treatment of fistula-in-ano, which used often to fail, has been placed on a better footing by Lockhart Mummery and a number of other workers. It is very helpful to have a law, which is nearly always correct, as to where to find the fistulous track and the internal opening; if the external opening is within $1\frac{1}{4}$ inches of the anus and in front, the internal orifice will be just opposite it; if it is farther away, or if it is anywhere behind the anus, the internal orifice will be in the mid-line at the back. If it is inevitable to cut the sphincter, a two-stage operation should be done, at the first laying the track open, and widely unroofed, starting from the external orifice, as far as the muscle but not through it; then later, when the parts have been made rigid by granulations, the sphincter is divided and the internal orifice laid open. Retraction of the ends of the muscle will be very slight if this technique is followed, and so the patient is not likely to suffer from incontinence. That troublesome condition, pruritus ani, sometimes fails to clear up with ordinary simple remedies, such as cleaning up with a sponge after defæcation, ointment, lotions, etc. I have been in the habit of operating by Ball's method, to denervate the peri-anal skin, and have been satisfied with the results. Lately I have been injecting the A.B.A. mixture used at St. Mark's Hospital, and it has acted very well.

We have now two good operations to choose from for cancer of the rectum, both, of course, severe, and both abandoning all hope of an anus in the natural position and leaving a permanent colostomy; but with a modern colostomy the patient is kept in reasonable comfort, and a considerable number of cases, as many as 50 per cent. in Lockhart Mummery's series, remain free from recurrence for five years. The one is the abdomino-perineal resection as described by W. E. Miles, which has a higher immediate mortality but is theoretically, at any rate, more likely to avert recurrence, and the other is colostomy followed by perineal excision. This is greatly facilitated, and can be carried a good deal higher up the bowel, by having the patient not in the lithotomy position but lying on his face in a sort of reversed Trendelenburg. I am indebted to Professor Wilkie for this valuable hint. But, of course, our hopes for the future are set upon the use of radium, and we trust the day is dawning when we can do without these formidable and mutilating procedures.

When the growth can only just be touched by the finger per rectum, and is too high for perineal excision, one can often, after a temporary colostomy made fourteen days previously, free the growth all round, resect it, and join the bowel end-to-end, over a rubber tube, below the peritoneal floor. Later the colostomy can be closed.

*Varicose Veins.*—Encouraged by the daily Press and by the puffing of quack practitioners, the public are greatly interested in the treatment of these by injection, and one finds large numbers of patients coming to have it done. I am very well pleased with the results. I use quinine-urethane as a rule, but in intractable cases sodium salicylate. It is needful, perhaps, to give a warning that even this simple and safe treatment can get one into dreadful trouble if a little solution escapes from a vein, and the veins are very soft and thin-walled sometimes. Also, occasionally, even large injections seem to cause no clotting at all. Still, the advantage that the patient can have his veins dissipated without going to bed is very acceptable.

*Treatment of Burns.*—There have been a good many "fashions" in the past in the treatment of burns. There was the Carron oil period, the eucalyptus and olive oil period, and the ambrine period. All these seem now definitely replaced by the introduction of the tanning method, which is greatly superior to all others. The patient is given an anæsthetic, and the burn cleaned up. It is then sprayed with tannic acid 2·5 per cent. by means of an ordinary atomizer, and this is repeated every hour until the whole of the burnt surface is tanned and black. This forms a firm protective covering which should be exposed to the air. The advantages of the method are that it is aseptic, nearly painless, inodorous, and, best of all, the dressing need not be changed for weeks at a time. Dressing a burn used always to be a dreadful process for all concerned. After a burn patients used to die either of shock on the first day or of toxæmia from scorch products a few days later, or of septic absorption later still. With the tanning treatment the second and third sources of fatality are eliminated, and deaths in hospital from burns are reduced from 50 per cent. to 10 per cent.

Burns on the face are best treated with absolute alcohol. The burn is covered with gauze and kept moist. The method is expensive, but leaves less scarring.

*Caries of the Spine.*—During the war I met an American surgeon who spoke warmly in favour of the Albee bone graft for tuberculous disease of the spine in adults, and as soon as I returned I gave it a trial, and was very gratified with the results. In properly selected cases, that is, not too recent, and not under sixteen, it is a very valuable method. It is not dangerous—it must not be confused with the much graver operation of laminectomy—and all my patients, some ten in number up to the end of 1927, have been enabled to get about and lead a fairly normal life, though some of them were paralysed, some had large dorsal or psoas abscesses, and some had been bedridden over four years. One recently climbed Ben Cruachan in Scotland.

*Sacro-iliac Tuberculosis.*—This used to be a very grave condition, almost a death-warrant, and the process of dying was long-drawn-out and miserable. Sir William Wheeler and others showed that it was capable of diagnosis before pus-formation, principally by the tenderness on direct pressure over the joint from behind, pain down the back of the leg especially on flexing the hip and extending the knee, and by the skiagram. The old text-book sign, pain on crowding the iliac crests together, is unreliable in early cases. And if diagnosed thus early, they also showed that it can be treated successfully by a simple and safe operation, by driving a bone-peg through the dorsum iliac, through the joint, into the lateral aspect of the sacrum. I have had quite a number of cases, considering that the disease is fortunately uncommon, and they have nearly all done well. When there are pelvic or gluteal abscesses it is too late for this treatment.

*Osteoarthritis of the Hip Joint.*—This can be a very painful, crippling condition in persons past middle life, and with no prospect of much relief either from time or treatment. But once again in properly-chosen cases, that is to say not too old or feeble to stand a fairly severe operation, and suffering from really considerable pain, usually with a badly-adducted thigh, great relief can be given by exposing the joint, chiselling away the whole articular surface of the head of the femur, and removing all bony outgrowths from the edge of the acetabulum. The limb can then be put up well abducted. The usual result is a painless, slightly mobile joint, in good position.

I fear, ladies and gentlemen, that this lecture has rather resembled the hectic career of a road-hog who whirls his party breathlessly past beautiful scenery that is well worthy of a more leisured view. I can only hope that you may have caught a fleeting glimpse of something that you will think sufficiently interesting to be looked into on a more peaceful occasion.

